# A combination of protein phosphatase 2A inhibition and checkpoint immunotherapy: a perfect storm

**DOI:** 10.1002/1878-0261.13687

**Published:** 2024-06-26

**Authors:** Mary C. Clark, Rongze Olivia Lu, Winson S. Ho, Matheus Henrique Dias, René Bernards, Stephen J. Forman

**Affiliations:** ^1^ Department of Hematology and Hematopoietic Cell Transplantation City of Hope Medical Center Duarte CA USA; ^2^ Department of Clinical and Translational Project Development City of Hope Medical Center Duarte CA USA; ^3^ Department of Neurological Surgery University of California, San Francisco CA USA; ^4^ Helen Diller Comprehensive Cancer Center University of California, San Francisco CA USA; ^5^ Division of Molecular Carcinogenesis, Oncode Institute The Netherlands Cancer Institute Amsterdam The Netherlands

**Keywords:** checkpoint inhibitor, PP2A, TIL therapy

## Abstract

Immune checkpoint blockade has emerged as a potent new tool in the war on cancer. However, only a subset of cancer patients benefit from this therapeutic modality, sparking a search for combination therapies to increase the fraction of responding patients. We argue here that inhibition of protein phosphatase 2A (PP2A) is a promising approach to increase responses to immune checkpoint blockade and other therapies that rely on the presence of tumor‐reactive T cells. Inhibition of PP2A increases neoantigen expression on tumor cells, activates the cGAS/STING pathway, suppresses regulatory T cells, and increases cytotoxic T cell activation. In preclinical models, inhibition of PP2A synergizes with immune checkpoint blockade and emerging evidence indicates that patients who have tumors with mutations in PP2A respond better to immune checkpoint blockade. Therefore, inhibition of PP2A activity may be an effective way to sensitize cancer cells to immune checkpoint blockade and cell‐based therapies using tumor‐reactive T cells.

## Graphical abstract

Inhibition of protein phosphatase 2A enhances immune destruction of cancer cells in two ways. First, in cancer cells, it leads to greater genomic instability and neoantigen expression. Second, in immune cells, it leads to the activation of cytotoxic T cells and inhibition of immunosuppressive regulatory T cells.

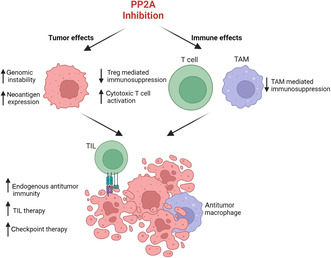

## 1 Introduction

Immunocheckpoint blockade (ICB) therapy has revolutionized cancer treatment. There are currently 11 checkpoint inhibitors approved by the Food and Drug Administration and international regulatory agencies [1], which act by increasing the activity of cytotoxic T cells against tumor cells. However, benefit from ICB therapy may be limited by impaired tumor infiltration by T cells, lack of tumor‐specific antigens, or an immunosuppressive tumor microenvironment (TME) [2]. Although there are ~90 approved indications for checkpoint inhibitors and more being evaluated in clinical trials, the greatest response has been in patients with tumors that are deficient in mismatch repair [1], as these tumors may express significant amounts of neoantigens. Identification of combination strategies to increase the effectiveness of ICB therapy is currently an area of active research [1]. Because ICB therapy relies on the presence of cytotoxic T cells that recognize tumor antigens, one approach is to combine checkpoint inhibitors with therapies that increase tumor antigen expression by increasing mutational burden. For instance, decreasing levels of mismatch repair protein MLH1 increases neoantigen generation and improves responses to ICB therapy in immunocompetent mouse models of cancer [3]. We discuss here the potential of protein phosphatase 2A (PP2A) inhibition to enhance responses to T cell‐mediated cancer therapy.

## 2 PP2A in cancer cells

PP2A acts on myriad pathways involved in tumorigenesis and cancer cell survival. Broadly speaking, PP2A negatively regulates cell proliferation and key survival pathways and therefore acts as a tumor suppressor [4]. Consistent with this function, downregulation of PP2A activity has been described in many cancers, including breast, ovarian, colorectal, and lung cancers [4]. PP2A is also involved in the DNA damage response and DNA repair pathways [5]. Thus, PP2A plays a role in maintaining genomic stability and preventing the accumulation of DNA damage. In cells that have decreased PP2A activity, double‐strand break repair is inefficient, rendering these cells susceptible to DNA damaging agents.

Because PP2A inhibits cell proliferation and survival and is downregulated in many cancers, there have been efforts to generate small‐molecule activators of PP2A. However, thus far, there are no PP2A activating agents being investigated in clinical trials. Another strategy is using PP2A inhibitors for cancers that retain PP2A activity, which may sensitize them to DNA damaging chemotherapy by inducing genome instability or DNA replication stress due to inhibition of DNA repair and increased mitogenic signaling [6]. One PP2A inhibitor, LB‐100, has been evaluated extensively in preclinical models of a variety of solid tumors and consistently enhances therapeutic efficacy of combination chemotherapeutic agents and radiotherapy [7, 8]. LB‐100 had a favorable safety and efficacy profile in a phase 1 dose‐escalation clinical trial to treat patients with relapsed solid tumors [7].

## 3 PP2A in immune cells

PP2A plays a major role in immune cells and regulates the type and magnitude of immune responses by influencing activation, differentiation, and exhaustion of immune cell populations [9, 10, 11, 12]. PP2A limits cytolytic function of T cells through negative regulation of NF‐kB signaling in response to T cell receptor engagement [10] and CTLA‐4‐mediated AKT signaling [13]. PP2A limits T cell activation by interacting with CARMA1; when dephosphorylated by PP2A, CARMA1 cannot recruit key mediators of T cell activation, leading to limited production of IL‐2 and IFN‐γ [12]. In an *in vivo* shRNA screen, silencing of a regulatory subunit of PP2A enhanced cytotoxic function of tumor‐infiltrating lymphocytes (TIL) in a melanoma model [14]. PP2A activity also impacts T cell differentiation through interaction with mTORC1 to promote regulatory T cell (Treg) differentiation and inhibit CD8+ cytotoxic T cells [9, 11]. Tregs have higher PP2A activity than conventional T cells, the reduction of which diminishes Treg suppressor function [9]. In macrophages, PP2A plays a role in regulating Toll‐like receptor‐mediated type‐I IFN and NF‐κB signaling in response to viral infections [15]. In tumor‐associated macrophages (TAMs), macrophage‐specific PP2A deficiency enhances cGAS‐STING‐mediated IFN production to promote antitumor immunity [16]. Overall, PP2A activity in immune cells contributes to immunosuppression by modulating innate and adaptive immune responses.

## 4 PP2A inhibition and tumor immunity

A robust tumor immune response requires a pool of T cells that are reactive to tumor neoantigens. However, cancer cells with high genomic stability typically have low neoantigen expression and may not elicit an immune response. Such cancers are typically referred to as immunologically “cold” and have low levels of TILs. Across multiple tumor types, the extent of genomic instability and/or tumor mutational burden are correlated with the number of TILs, which may influence therapeutic response and survival outcomes [17]. Therefore, increasing tumor neoantigens by increasing genomic instability (i.e., going from immunologically “cold” to “hot”) represents a method of increasing the population of tumor‐reactive cytotoxic T cells.

Pharmacologic inhibition of PP2A increases neoantigen expression by altering mRNA splicing [18] and increases microsatellite instability by epigenetic silencing [19]. In colorectal adenocarcinoma cells, LB‐100 treatment causes exon skipping and increased alternative splicing, potentially contributing to neoantigen expression [18]. In glioblastoma cells, PP2A inhibition enhances cytosolic double‐stranded DNA production to promote cGAS‐STING‐IFN signaling in tumor and immune cells [20]. Moreover, inhibition and/or genetic ablation of PP2A sensitize cancer cells to ICB therapy in solid tumor models [19, 21]. Therefore, PP2A inhibition may enhance the tumor immune response through multiple complementary mechanisms: (a) increasing genomic instability and neoantigen expression, thereby increasing the pool tumor‐reactive TILs; (b) increasing T cell activation; (c) skewing toward a less tolerant, more effector T cell phenotype; and (d) reducing the number of immunosuppressive macrophages within the TME.

## 5 Combining PP2A inhibition with immune‐based therapies

**Fig. 1:**
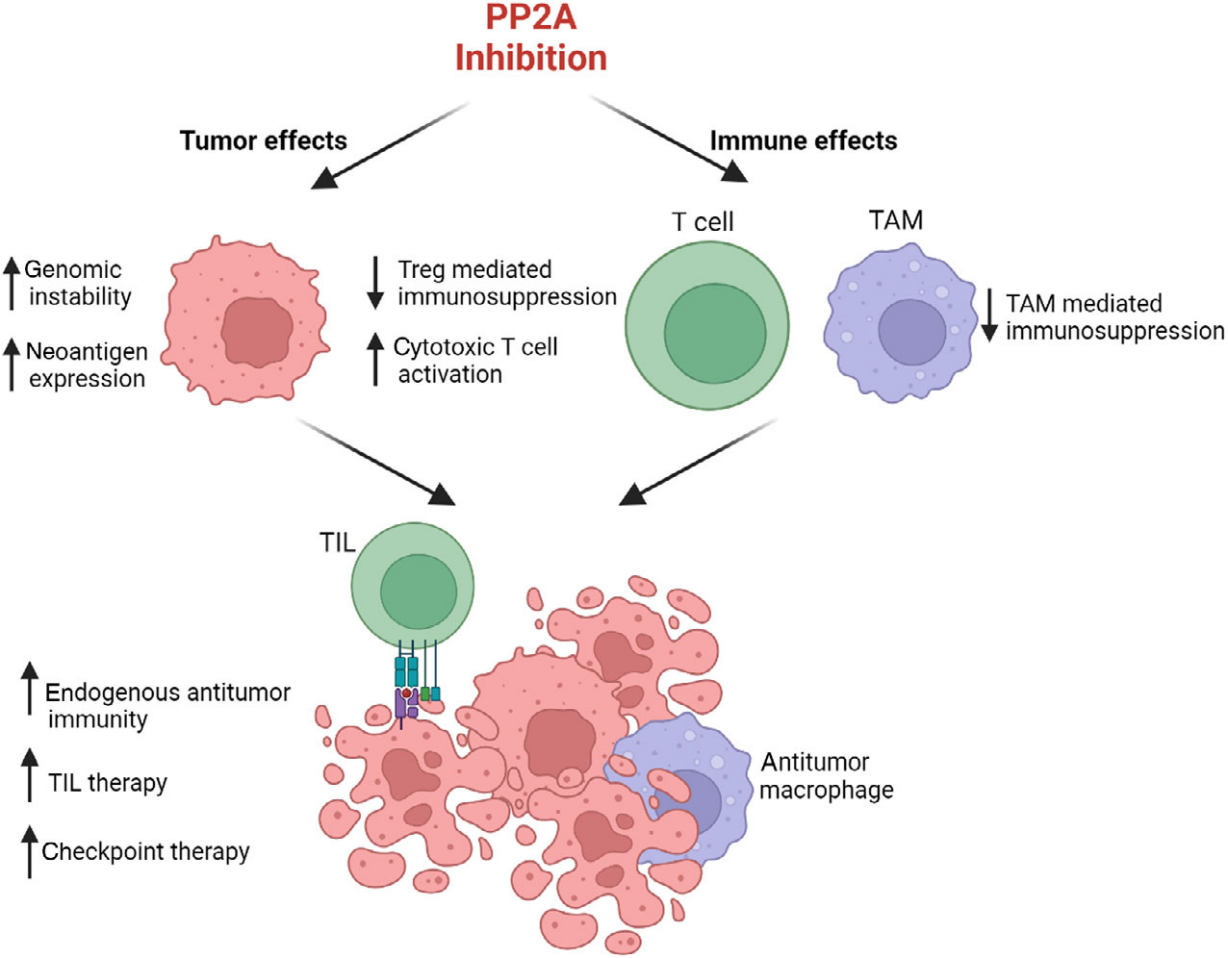
PP2A inhibition may enhance the efficacy of immune‐based cancer therapies. PP2A inhibition affects both cancer cells and immune cells. In cancer cells, PP2A enhances neoantigen expression in part by promoting alternative RNA splicing and genomic instability. By increasing the number of targets that are recognizable by endogenous immune cells, PP2A inhibition may increase the number of tumor‐infiltrating lymphocytes (TIL). In immune cells, inhibiting PP2A enhances immune activation through many mechanisms, including decreasing the number and immunosuppressive activity of both regulatory T cells (Tregs) and tumor‐associated macrophages (TAMs). PP2A inhibition also increases the activation of cytotoxic T cells. Therefore, PP2A inhibition increases the endogenous antitumor immune response, which may be leveraged for combination strategies that require an endogenous pool of tumor‐reactive T cells, including TIL therapy and checkpoint therapy. Figure generated in BioRender.com.

Given that PP2A inhibition is well‐tolerated in patients with solid tumors and may modulate immune reactivity, it is attractive to combine PP2A inhibition with immune‐based therapeutic strategies, including TIL and ICB therapies (Fig. 1). Both TIL and ICB therapies rely on the capacity of T cells to recognize and target tumor cells. Expanding the repertoire of tumor antigens available for immune recognition by inhibiting PP2A is, therefore, one method to potentially improve activity of these therapies. Moreover, PP2A inhibition has the potential to extend immune‐based therapies to cancers that are typically considered genomically stable and immunologically cold, including ovarian cancer [22]. Thus, by affecting both cancer cells and immune cells, PP2A inhibition may improve immune‐based therapies for cancer.

In a recent retrospective analysis of ovarian clear cell carcinoma [23], the authors investigated whether specific mutations, including those in PP2A, were associated with survival outcomes following ICB therapy. In the 28‐patient cohort, approximately 25% had an inactivating mutation in PP2A, which was associated with statistically better survival outcomes in the context of ICB therapy [23]. A similar conclusion was reached by retrospective analysis of a large cohort of cancer patients treated with ICB therapy [24]. Although the mechanism behind this therapeutic benefit is unknown, low PP2A activity may have influenced the generation of tumor‐reactive T cells for the checkpoint inhibitor to exert its function by increasing tumor neoantigen expression. While the causal relationship between loss‐of‐function PP2A mutations and better survival in the context of ICB therapy is likely complex and multifactorial, we pose here that PP2A activity is one factor that may be leveraged to enhance the effectiveness of ICB therapy. Moreover, it is possible that inhibiting PP2A pharmacologically may mimic an inactivating mutation, increase the pool of tumor‐reactive T cells as well as the capacity for T cell activation, and potentially increase the efficacy of checkpoint therapy for the treatment of cancer. Indeed, in preclinical models, inhibition of PP2A has shown significant synergy with PD1 therapy [19, 21, 25].

Adoptive cellular therapy using TILs isolated from individual patients is promising for specific cancers, including metastatic melanoma [17]. In this therapeutic modality, tumor‐specific T cells are isolated, expanded *ex vivo*, and infused into the patient. Thus, TIL therapy requires a preexisting pool of endogenous cytotoxic T cells that recognize tumor antigens and works best in context of cancers with high mutational burden and T cell infiltration. Response to TIL therapy, alone or combined with other therapies, for other solid tumor types has been modest, with overall response rates of 8–50% [17]. Lack of response to TIL therapy is partly due to the relatively low mutational burden and infiltration of T cells into nonmelanoma solid tumors. Thus, novel approaches to increase the pool of tumor‐reactive T cells are needed to increase the potential use of TIL therapy, and we argue that pharmacologic inhibition of PP2A represents one such approach. PP2A inhibition may have the additional benefit of releasing constraints on T cell activation, skewing T cell subsets toward reactivity, and increasing the capacity of infiltrating immune cells to kill tumor cells.

## 6 Conclusion

Modulating PP2A activity may confer therapeutic benefits by directly affecting cancer cells as well as through immunologic effects. PP2A inhibition may augment the tumor immune response by increasing neoantigens, increasing the number and activity of tumor‐reactive cytotoxic T cells and decreasing immunosuppression by Tregs and tumor‐associated macrophages [TAMs] in the TME. We acknowledge that this is likely not the whole answer to increasing the effectiveness of immune‐based therapies for cancer, especially when cancer cells are PP2A deficient. Nevertheless, increasing tumor‐specific targets for the immune system is a possible step forward. Thus, PP2A inhibition would likely improve the activity of immune‐based therapies and should be pursued as a rational combination therapeutic approach. Currently, two active clinical trials are evaluating immune checkpoint inhibitors in combination with PP2A inhibition (NCT04560972 and NCT06065462).

## Conflict of interest

SFJ is a member of the board of directors of Lixte and Allogene Therapeutics and has received grant support from MustangBio. RB is a member of the board of directors of Lixte, received research funding from Lixte, and is a shareholder of Lixte. MCC, ROL, and WSH have no conflicts to disclose.

## Author contributions

MC, RL, WH, MD, RB, and SF conceived and designed the project and wrote the manuscript. SFJ is a member of the board of directors of Lixte and is a scientific advisor to Allogene Therapeutics and MustangBio. RB is a member of the board of directors of Lixte, received research funding from Lixte, and is a shareholder of Lixte. MC, RL, MD, and WH have no conflicts to disclose.
